# Mitochondrial Oxidative and Nitrosative Stress and Alzheimer Disease

**DOI:** 10.3390/antiox9090818

**Published:** 2020-09-02

**Authors:** D. Allan Butterfield, Debra Boyd-Kimball

**Affiliations:** 1 Department of Chemistry and Sanders-Brown Center on Aging, University of Kentucky, Lexington, KY 40506, USA; 2 Department of Chemistry and Biochemistry, University of Mount Union, Alliance, OH 44601, USA; boydkidl@mountunion.edu

**Keywords:** oxidative and nitrosative stress, mitochondrial dysfunction, Alzheimer disease and mild cognitive impairment, redox proteomics, amyloid beta-peptide

## Abstract

Oxidative and nitrosative stress are widely recognized as critical factors in the pathogenesis and progression of Alzheimer disease (AD) and its earlier stage, amnestic mild cognitive impairment (MCI). A major source of free radicals that lead to oxidative and nitrosative damage is mitochondria. This review paper discusses oxidative and nitrosative stress and markers thereof in the brain, along with redox proteomics, which are techniques that have been pioneered in the Butterfield laboratory. Selected biological alterations in—and oxidative and nitrosative modifications of—mitochondria in AD and MCI and systems of relevance thereof also are presented. The review article concludes with a section on the implications of mitochondrial oxidative and nitrosative stress in MCI and AD with respect to imaging studies in and targeted therapies toward these disorders. Taken together, this review provides support for the notion that brain mitochondrial alterations in AD and MCI are key components of oxidative and nitrosative stress observed in these two disorders, and as such, they provide potentially promising therapeutic targets to slow—and hopefully one day stop—the progression of AD, which is a devastating dementing disorder.

## 1. Introduction

Alzheimer disease (AD) is the single largest cause of dementia in the aged population. In the United States, nearly six million persons have AD, with projections of between 15–20 million persons predicted to have AD within 30 years due to the aged-related nature of AD coupled to the very large Baby Boomer population in the USA (estimated to be up to 75 million Americans) [1]. The human and financial costs of this level of AD patients, in the opinion of the authors, make the future of AD a public health crisis for the USA and other countries that will require science-based interventions to slow the development or progression of AD [1].

The principal pathological hallmarks of brain in AD have been well described, i.e., extraneuronal deposits of fibrillar amyloid beta-peptide (Aβ) and dystrophic neurites (senile plaques, SP), intracellular accumulations of neurotoxic Aβ oligomers, intracellular deposits of hyperphosphorylated tau (neurofibrillary tangles, NFT), with tau being a key microtubule stabilizing protein, and synapse loss [2,3,4]. However, the complete molecular basis of AD remains elusive. Investigators have demonstrated that cognitive decline correlates well with Aβ oligomer and NFT loads, but not SP levels [2,3,4]. Consistent with the lack of correlation of SP with cognitive loss, therapeutic attempts to remove SP largely have not proven successful in modifying the course of AD [5]. An earlier stage of AD, amnestic mild cognitive impairment (MCI), is characterized by memory loss validated by an independent observer with otherwise normal activities of daily living [6]. Pathology in MCI is similar to that in AD, albeit to a somewhat lesser extent [7].

Other factors may be involved in the progression of cognitive loss in AD. For example, oxidative and nitrosative stress in the brain are now widely recognized as key aspects of the pathogenesis and progression of AD and MCI [8,9,10,11,12]. Oxidative and nitrosative stress arise when the production of oxygen- or nitrogen-containing free radicals or reaction products of these two moieties exceed the cellular capacity to scavenge such species. As mentioned below, mitochondria are a major source of reactive oxidative stress and are involved in the production of nitrosative stress as well [8,13].

Consequently, this review will focus on mitochondria and oxidative and nitrosative stress in AD and MCI.

## 2. Mitochondria and Oxidative and Nitrosative Stress

Mitochondria are the principal source of ATP, which is formed by oxidative phosphorylation following electron flow through highly integrated protein complexes; however, mitochondria are also involved in many other functions as well, which, among others, include apoptosis, protein synthesis, and fatty acid oxidation [14,15].

**Figure 1:**
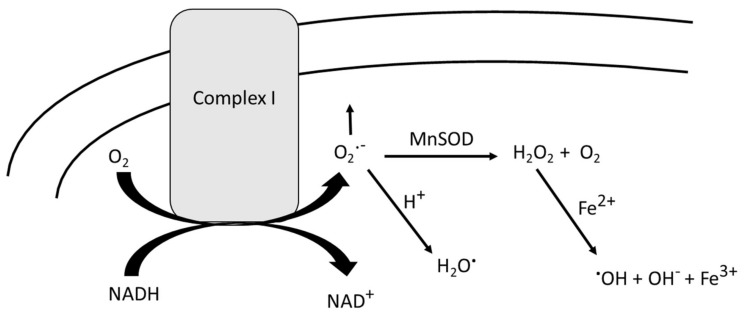
In the process of oxidizing NADH by mitochondrial Complex I, oxygen is partially reduced to superoxide free radical, and a small percentage of leaks out. The protonation of superoxide radical anion leads to a non-charged free radical that can both easily penetrate and even cross the inner membrane lipid bilayer. Matrix-resident manganese superoxide dismutase (MnSOD) catalyzes the disproportionation of superoxide free radical into hydrogen peroxide and oxygen. H_2_O_2_ can react with any adventitious Fe^2+^ or Cu^+^ to form the highly reactive hydroxyl free radical, which causes oxidative damage to any nearby protein or lipid. Moreover, H_2_O_2_ has a zero dipole moment, giving this molecule a non-polar character, which allows penetration into the lipid bilayer where lipid peroxidation may ensue. See the text for more details.

Electrons supplied by reducing equivalents produced by glycolysis and the tricarboxylic acid cycle interact with Complex I of the electron transport chain (which in the case of NADH requires elaborate transport channels, since NADH cannot cross the inner membrane to the matrix). As electron flow occurs in the multiple redox-sensitive proteins within Complex I on the inner membrane, electron leak occurs, usually in the form of superoxide radical anion or the hydrogendioxide radical, elevating oxidative stress (Figure 1) [8,16,17]. Normally, superoxide free radicals are neutralized by mitochondrial matrix-resident manganese superoxide dismutase (MnSOD), producing hydrogen peroxide (H_2_O_2_) and molecular oxygen [16]. The former can be decomposed by catalase, but while this is an extraordinarily efficient enzyme it is often packaged in such a way as not to readily decompose this molecule. In that case, H_2_O_2_, which has zero dipole moments and is therefore neutral and can diffuse into or through bilayers, can be decomposed to hydroxyl anion and the highly reactive hydroxyl free radical by Fe^2+^ or Cu^+^ in a process known as Fenton chemistry [8,13].

Other mitochondrial-related antioxidant systems include, among others, thioredoxin [18], peroxiredoxin 4 [19], glutathione peroxidase 4 [20], and methionine sulfoxide reductase A [21]. These enzymes play essential roles in maintaining redox homeostasis in mitochondria by exerting critical redox protective effects, including against protein oxidation. Consequently, dysfunctions in the activities of these enzymes are detrimental to cell survival.

**Figure 2:**
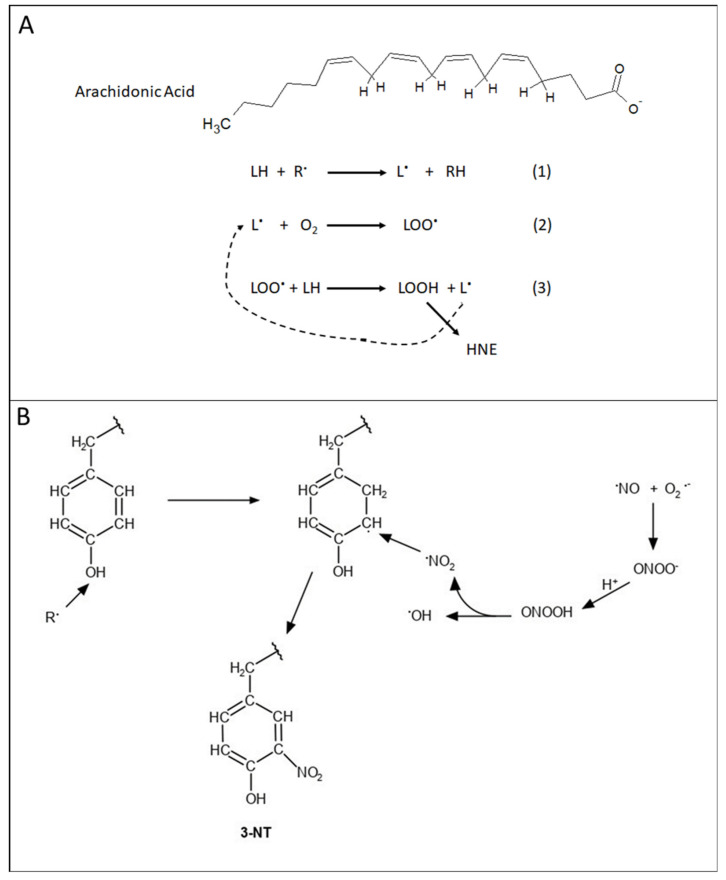
(**A**) Mechanism of lipid peroxidation. Arachidonic acid structure showing labile allylic H-atoms that are subject to free radical-mediated abstraction (Rxn 1) leading to carbon-centered free radical on the lipid-resident arachidonic acid carbon backbone. Paramagnetic molecular oxygen, in a radical–radical recombination reaction (Rxn 2), produces the lipid-bound peroxyl free radical. This latter free radical abstracts another labile allylic H-atom (Rxn 3) to form the lipid hydroperoxide and another carbon-centered free radical. Then, this latter radical takes part in Rxn 2 again, i.e., a chain reaction, that will continue as long as there are molecular oxygen and allylic H-atoms present. The lipid hydroperoxide is decomposed into reactive aldehydes, including the highly reactive 4-hydroxynonenal (HNE). See text for additional details. (**B**). Formation of 3-nitrotyrosine. Nitric oxide (NO), a free radical produced by nitric oxide synthase from arginine, reacts with superoxide free radical anion (see Figure 1) by radical–radical recombination to form peroxynitrite, ONOO^−^. The actual next reaction shown is more complicated than depicted, but in essence, the protonation of peroxynitrite forms peroxynitrous acid that decomposes into the free radical nitrogen dioxide (NO_2_) and hydroxyl free radical. Tyrosine, attacked by a free radical R^.^ to remove the H-atom of the phenolic OH group, leaves an unpaired electron on the O-atom that delocalizes to the 3-position of tyrosine with the H-atom removed from the 3-position, reforming the OH functional group on Tyr. NO_2_ reacts with the delocalized unpaired electron on the 3-position of tyrosine residues in a radical–radical recombination reaction to form 3-nitrotyrosine (3-NT), which is a major marker of nitrosative stress. See the text for further details.

Hydroxyl radical readily reacts with proteins and allylic H-atoms on the fatty acid tails of phospholipids and sphingolipids. The former leads to the incorporation of carbonyl functionalities in proteins and alterations in protein conformation (and normally loss of function as a consequence) [8,22,23,24], while OH-mediated allylic hydrogen atom abstraction from unsaturated lipid acyl chains initiates the processes of lipid peroxidation, leading to highly reactive nucleophilic unsaturated aldehydes such as 4-hydroxynonenal (HNE) (Figure 2A) [8,13,25,26,27]. Protein conformation is also altered with loss of function by HNE that binds to and changes the structure of proteins [28].

Nitrosative stress usually follows the production of nitric oxide (NO), which is formed by the decomposition of arginine catalyzed by the enzyme nitric oxide synthase (NOS) [29]. In the brain, neuronal NOS is involved in glutamatergic neurotransmission processes and requires Ca^2+^. Inducible NOS (i-NOS) does not require Ca^2+^ and is key to nitrosative stress in the brain. When superoxide radical anion (for example from mitochondria) and NO (also a free radical) meet, their reaction rate is extremely high, and in the presence of CO_2_ leads to the formation of nitrogen dioxide (NO_2_) [29]. The formation of 3-nitrotyrosine (3-NT) on proteins occurs when the phenolic H-atom of tyrosine is removed by a radical attack followed by electron delocalization to the 3-position of Tyr and radical–radical recombination of this radical with NO_2_ occurs (Figure 2B). With the NO_2_ functionality on Tyr, tyrosine phosphorylation by tyrosine kinases, including receptor tyrosine kinases, is inhibited due to steric interference of the NO_2_ group with the active site of the enzyme. This modification can provide detrimental consequences for cell survival [8,13,29,30].

## 3. Selected Biological Alterations in AD Mitochondria

While mitochondria have a principal function of producing ATP, other functions also are important and relevant to AD. As one example, elegant research from Mahley’s laboratory demonstrated in Neuro-2a neuronal cells expressing stable isoforms of apolipoprotein E (apoE), i.e., apoE2, apoE3, or apoE4, with the latter a major risk factor for developing AD, that apoE4 is excessively produced by damaged neurons [31]. Since the structure of apoE4 is different, due to the lack of Cys residues, compared to two key Cys residues in apoE2 and one key Cys residue in apoE3 [32], apoE4 is subject to proteolysis. The resulting apoE4 fragments were demonstrated by the Mahley laboratory to be neurotoxic, specifically negatively affecting tau biology and mitochondrial function [31]. For the latter, mitochondrial respiration (as well as glycolysis) was negatively affected under basal conditions, but if stressed or damaged, apoE4 neurons had half the ATP reserve capacity of apoE3 neurons. A lower NAD^+^/NADH ratio and elevated ROS and mitochondrial Ca^2+^ also were observed. These changes were correlated to proteomics-identified altered levels of proteins associated with mitochondrial fission/fusion dynamics among other mitochondrial proteins [31]. The researchers suggested that important therapeutic targets for AD emerged from these studies.

The lack of Cys residues in apoE4 has been hypothesized to contribute to the increased risk of developing AD, since the lipid peroxidation product HNE would not be able to be scavenged (unlike the case for apoE2 and apoE3) and thereby bind to and modify the structure and function of mitochondrial proteins in AD and MCI [33]. It is conceivable that the studies of Mahely [31] and Butterfield [33] have significant overlap, although other studies would be required to test this notion.

Consonant with the above studies, Swerdlow [34] posited the existence of primary and secondary mitochondrial cascades as critical for AD. Primary mitochondrial cascades in AD are hypothesized to be due to diminished glucose metabolism in an AD brain and in tissues outside the brain. The latter is suggested to indicate that Aβ peptide is not needed for mitochondrial dysfunction. The secondary mitochondrial cascade important in AD posited by Swerdlow involves Aβ oligomer-induced changes in mitochondrial function, as well the presence of a dehydrogenase, amyloid-binding alcohol dehydrogenase (ABAD), in mitochondria, that leads to elevated free radicals, consequent altered glycolytic and mitochondrial proteins structure and function, and resultant Ca^2+^ elevation, causing neuronal death. As Swerdlow suggests, both mitochondrial cascades could be occurring simultaneously [34].

In a recent reviews and discussed further below, evidence was collected for altered glucose metabolism associated with Aβ-mediated lipid peroxidation and protein oxidative damage to glycolytic and mitochondrial enzymes, ion-motive ATPases, the opening of voltage-gated Ca^2+^ channels with resulting Ca^2+^ elevation in endoplasmic reticulum and mitochondria, and consequent neuronal death [8,35]. This would suggest support for the Secondary Mitochondrial Cascade hypothesis for AD. Moreover, mitochondria isolated from lymphocytes from persons with AD and MCI showed elevated oxidative damage that correlated inversely with cognitive performance [36,37]. Redox proteomics identified similar proteins as identified in AD and MCI brains as oxidatively modified, and these included mitochondrial ATP synthase [36,37]. Consequently, evidence consistent with the tenants of the Primary Mitochondrial Cascade hypothesis were obtained, suggesting, similar to that posited by Swerdlow, both cascades are in play in AD and MCI that coalesce in the brain as decreased glucose metabolism evidenced by altered glycolysis and mitochondria. This concept is discussed further below.

Mitochondria are not static organelles. For example, anterograde and retrograde axonal transport of mitochondria by kinesin and dynein, respectively, along microtubule assemblies are needed to provide pre-synaptic membranes, vesicles, etc. with sufficient ATP to permit neurotransmission and regulate neuronal function and to return to the neuronal cell body to be recharged. Other dynamics in which mitochondria are involved are fission and fusion. Among other functions, mitochondrial fission is involved in the regulation of mitophagy, apoptosis, and the cellular need for ATP [38]. In contrast, mitochondrial fusion leads to large moieties that combine contents of multiple mitochondria, which also leads to pleiotropic functions, among which is a larger assembly of mitochondrial DNA that is needed to produce a subset of mitochondrial proteins [38]. The role of dynamin-related protein-1 (Drp-1) in these processes has been extensively investigated [39]. Drp-1 is critically important for mitochondrial fission, but when its expression is lowered, there is some evidence that Drp-1 may play a role in mitochondria fusion as well [39]. Drp-1 is a GTPase, and the enzyme activity allows Drp-1 to dissociate from the receptor that was ligand-activated. This GTPase is highly susceptible to post-translational modification (PTM). Among the various PTMs, S-Nitrosylation of the GTPase activity region of Drp-1 leads to a loss of activity and in a mutant APP/PS1 transgenic mouse model of AD, it leads to neurotoxicity [39]. The inhibition of mitochondrial fission with mitochondrial division inhibitor-1 (Mdivi-1), which indirectly inhibited Drp-1, protected against the production of reactive oxygen species (ROS), loss of anterograde mitochondrial transport, damage to synapses, neuronal death, and stunted growth of neuritic spines, all of which were observed in untreated mice [40,41]. Decreased mitochondrial fragmentation was observed in vitro using N2a neuronal cells treated with Mdivi-1 prior to challenge with neurotoxic Aβ [42].

## 4. Redox Proteomics

Redox proteomics is a subset of proteomics methods used to identify oxidatively or nitrosatively modified proteins [43]. The authors of this section of the review focus on redox proteomics applied to studies of relevance to AD, MCI, and preclinical AD. Redox proteomics was pioneered in the Butterfield laboratory [44], and the reader is directed to comprehensive reviews of redox proteomics applied to neurodegenerative disorders and AD specifically [45,46,47,48,49,50,51,52], as well as a recent review of redox proteomics applied to studies of Aβ in in vitro and in vivo investigations and in studies of AD and MCI [35]. 

Redox proteomics adopts many of the methods of expression proteomics, i.e., separation of proteins of interest, i.e., those that are oxidatively or nitrosatively modified (indexed by protein oxidation employing protein-resident carbonyls and 3-NT, or lipid peroxidation, indexed by protein-bound HNE). These proteins are modified by appropriate tagging strategies to identify subject versus control proteins, which is followed by the digestion of proteins with trypsin and the separation of tryptic peptides often through ion-exchange columns followed by reverse-phase chromatography, with each tryptic peptide injected into a high-response mass spectrometer operating in MS/MS mode. This approach allows for the amino acid sequence of separated peptides to be determined, from which the identification of the proteins involved can be determined by interrogating various databases. The relative heights of mass peaks of each peptide from control and AD brain specimens, with different markers on the control and AD peptide being examined, give the investigator knowledge of the relative abundance of the proteins of interest. For much more detailed descriptions of the methods involved in redox proteomics, the reader is referred to a descriptive analytical review paper [46].

As will be further described below, the application of these methods to in vitro cellular models, in vivo animal models, and human brain from AD and MCI and respective control subjects, with emphases on mitochondrial proteins identified will be the subject of the remaining aspects of this review paper. An important point is that in all studies from our laboratory at the University of Kentucky, post-mortem intervals of brain from which specimens were obtained were typically in the range of 2–4 h. Any longer times, in our opinion, would render interpretations of redox proteomics results as less than reliable for obvious reasons.

## 5. Oxidative and Nitrosative Modifications of Mitochondrial Proteins in AD and MCI and in Systems of Relevance Thereof

As mentioned above, the use of redox proteomics has led to the identification of oxidized proteins, as indexed by carbonylation, nitration, and modification with HNE, in both in vitro and in vivo models of AD in addition to MCI and AD brain. Consistent with the hypothesis that both oxidative stress and mitochondrial dysfunction are early events in the progression of AD [12,35,53], many of the oxidized proteins identified are mitochondrial proteins involved in metabolism, protein synthesis and folding, ROS scavenging, transport, and apoptosis (Table 1).

**Figure 3:**
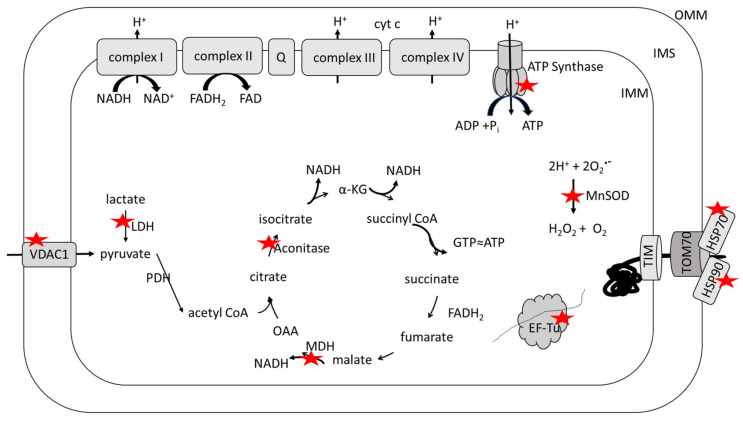
Redox proteomics identified oxidatively modified, mitochondrial proteins in brains from persons with Alzheimer disease or amnestic mild cognitive impairment. Proteins marked with a red star are oxidatively modified. Functional classes of oxidatively dysfunctional proteins include those related to: Glucose metabolism, i.e., lactate dehydrogenase (LDH), aconitase, malate dehydrogenase (MDH), ATP synthase; Export of anions (ATP, for example, has a decreased chance of export through voltage-dependent anion channel 1 (VDAC1) to drive neuronal function including the maintenance of mitochondrial potential); Free radical scavenging (MnSOD, which increases opportunities to cause further oxidative and nitrosative damage to mitochondria); mitochondrial protein synthesis (EF-Tu, which would lead to altered levels of key proteins needed to assemble into functional inner membrane resident electron transport complexes and thereby decrease ATP levels); and chaperone and transport function for the import of nuclear-encoded proteins into mitochondria (heat shock protein 70 (HSP70); heat shock protein 90 (HSP90), which also could contribute to altered protein levels of needed electron transport chain [(ETC) complexes and other key proteins]. See text for further details.

Several proteins associated with catabolism and mitochondrial ATP generation are oxidized in MCI and AD brains (Figure 3). These include the tricarboxylic acid cycle enzymes aconitase in late AD [54] and malate dehydrogenase (MDH) in early AD [55] and the alpha-subunit of ATP synthase, or complex V of the electron transport chain, in MCI [56], early AD [55,61], and late AD [54,57]. Aconitase is an iron–sulfur protein that catalyzes the interconversion of citrate and isocitrate [62] and is a redox-sensitive enzyme that is significantly modified by HNE in late AD hippocampus [54] and by carbonylation induced by Aβ in vivo [63]. MDH catalyzes the oxidation of malate to oxaloacetate with the reduction of NAD^+^ to NADH, but it also plays a critical role in the malate–aspartate shuttle that maintains the redox status of the cytosol by shuttling electrons from NADH in the cytosol to NAD^+^ in the mitochondrial matrix [64]. MDH is oxidatively modified by HNE in early AD inferior parietal lobule (IPL) [55] and its carbonylation is induced by Aβ in vitro and in vivo [63,65,66]. Lactate dehydrogenase-B (LDH) is also present in mitochondria [67] and is oxidatively modified by HNE in MCI hippocampus [56]. LDH is found preferentially in aerobic tissues [68] and allows neuronal mitochondria to utilize lactate imported by monocarboxylate transporter 2 (MCT2) as a fuel source by oxidation to pyruvate from which acetyl CoA can be produced and condensed into the tricarboxylic acid cycle at citrate synthase [67,69,70]. ATP synthase utilizes the protein gradient established across the inner mitochondrial membrane by the electron transport chain to drive the phosphorylation of ADP to yield ATP [71,72]. The carbonylation of ATP synthase is induced in vivo by Aβ [73], and it is significantly modified by HNE in MCI hippocampus and IPL [56], early AD IPL [55], and late AD IPL [54] in addition to being significantly nitrated in late AD hippocampus [57]. The activity of each of these oxidatively modified enzymes is affected in MCI and AD brains. The activities of LDH, aconitase, and ATP synthase are significantly decreased as a result of oxidation [54,55,56,61] and consistent with the hypometabolism prevalent in MCI and AD brain [74,75,76]; however, the activity of MDH is significantly increased [55]. It is unclear why the oxidative modification results in structural change that increases the activity of MDH; however, the increased activity could be a compensatory mechanism in response to the oxidative damage of other key energy-related proteins including a number of glycolytic enzymes [35].

Proteostasis is the maintenance of the proteome ensuring that all proteins are correctly folded, localized, and concentrated for their function. Mitostasis refers specifically to mitochondrial proteostasis [77]. Mitochondria possess both circular DNA and a transcription–translation machinery [78]. EF-Tu is a mitochondrial elongation factor responsible for coordinating the binding of amino acyl-tRNA to the codon [79] and is significantly modified by the lipid peroxidation product HNE in MCI IPL [56]. The oxidation of EF-Tu could lead to altered function and the incorrect translation of mitochondrial proteins, which include 13 components of the electron transport chain [78].

While mitochondria possess their own DNA and transcription and translation machinery, most mitochondrial proteins are encoded in nuclear DNA, and the protein product needs to be transported into mitochondria from the cytosol in an unfolded state [77]. Heat shock protein 70 (HSP70) and heat shock protein 90 (HSP90) are chaperones that interact with the outer mitochondrial membrane, stabilizing the unfolded state of the nascent proteins and thereby preventing aggregation, and they are required for transport of the protein into mitochondria through the translocase Tom70 [80]. Both HSP70 and HSP90 are significantly carbonylated in MCI IPL [59,60], while HSP70 is modified by both HNE and 3-NT in an MCI hippocampus [56,58]. Oxidation of these chaperones may prevent the formation of appropriate interactions with protein targets, resulting in protein misfolding and aggregation, thereby impacting the transport of nuclear encoded proteins into mitochondria.

MnSOD, or superoxide dismutase 2 (SOD2), is a homotetramer localized to the mitochondrial matrix. MnSOD catalyzes the conversion of superoxide radical anion into H_2_O_2_ and O_2_ [81,82,83], and its expression is increased in AD hippocampus [84]. MnSOD is oxidatively modified by HNE in early AD IPL, resulting in a significant decrease in enzyme activity [55] and is also oxidatively modified by HNE in late AD IPL [54]. Decreased activity of MnSOD would result in a decreased capacity to scavenge superoxide radical anion propagating free radical damage to mitochondrial components resulting in further mitochondrial dysfunction. MnSOD activity is suppressed by the binding of p53 [85]. p53 is a nuclear transcription factor that translocates to the mitochondria as a result of stress, including ROS, leading to apoptosis and necrosis. Translocation leads to an accumulation of p53 in the mitochondrial outer membrane and binds to inhibitors of apoptosis proteins, Bcl-2 or Bcl-xL, which releases Bax and Bak and thereby contributes to the formation of a mitochondrial permeability transition pore (MPTP) and release of cytochrome c. p53 can also accumulate in the inner mitochondrial membrane and mediate the opening of MPTP in the inner mitochondrial membrane [86]. p53 is significantly increased in MCI and AD IPL with p53 significantly modified by protein-bound HNE, 3-NT, and carbonyls in an AD brain. p53 is significantly modified by carbonylation, but not 3NT or HNE in MCI IPL [87,88]. Protein expression of MnSOD was significantly increased in mitochondria isolated from the brain of p53 knockout mice [89], which is consistent with the p53 transcriptional repression of MnSOD [90,91]. This relationship is suggested to fine-tune the cellular response to oxidative and nitrosative stress [92], but oxidative damage to MnSOD and p53 in MCI and AD brain may affect this interplay, which contributes to apoptosis [93] and to the spread of ROS and subsequent oxidative damage beyond the subcellular localization of the mitochondria.

The voltage-dependent anion channel 1 (VDAC1), located in the outer mitochondrial membrane, regulates mitochondrial membrane permeability [94] and is significantly nitrated in late AD hippocampus [57]. VDAC1 regulates the entry of metabolites (i.e., pyruvate, malate, and succinate) and ions in addition to the exit of hemes and ROS. Thus, VDAC1 mediates communication and traffic between mitochondria and the cytosol [95]. The N-terminal domain of VDAC1 interacts with several Bcl-2 family proteins that regulate apoptosis and is involved in gating the channel [96,97,98,99,100,101]. A dynamic equilibrium between monomeric and oligomeric states of the protein exist, with the oligomeric state being formed upon apoptotic induction resulting in a channel through which cytochrome c efflux can occur [94]. VDAC1 inhibitors prevent oligomerization and the induction of apoptosis even in the presence of pro-apoptotic stimuli [102], indicating that apoptotic induction can be driven by VDAC1 oligomerization. VDAC1 overexpression, which favors oligomerization and promotes apoptosis [103], is present in AD brain and increases as the disease progresses [104]. The hexokinase isoform HK-I binds to the N-terminus of VDAC1 on the cytoplasmic side of the outer mitochondrial membrane and has been shown in both in vitro and in vivo systems to protect against apoptosis [105,106]. Aβ(1–42) also directly interacts with the N-terminus of VDAC1 and induces HK-I release, resulting in VDAC1 oligomerization, cytochrome c release, and apoptosis in SH-SY5Y cells [107]. The oxidative modification of VDAC1 by 3-NT in AD brain could induce apoptosis by affecting the structure of the protein to either promote HK-1 release or oligomerization directly. Further study is needed to distinguish between these possibilities.

Taken together, the oxidative modification of proteins critical for maintaining cellular energy and redox status, metabolite, and ion transport, the translation of mitochondrial encoded proteins, and the import of nuclear-encoded proteins could play a critical role in the mitochondrial dysfunction observed early in the pathogenesis and progression of AD.

Oxidative stress has also been characterized in mitochondria isolated from peripheral cells in MCI and AD. Levels of protein-bound carbonyls, 3-NT, and HNE are significantly increased in mitochondria isolated from lymphocytes of MCI and AD patients, which correlate with elevated Aβ levels, decreased levels of small molecule antioxidants, and decreased cognitive test performance [36,37]. Further study using quantitative proteomics identified protein expression alterations in mitochondria isolated from both MCI and AD lymphocytes. These proteins can be categorized as involved in energetics, structural integrity, cell signaling, and antioxidant defense [37]. Interestingly, significant increases in all the proteins affected in MCI lymphocyte mitochondria were detected. Notably, these proteins included LDH-B and the β-subunit of ATP synthase, indicating an upregulation to meet the energy needs of the cells early in the disease process that does not persist into the development of AD. Consistent with a possible compensatory mechanism in the progression of the disease is a significant increase in the activity of electron transport chain complexes II and IV reported in mitochondria isolated from AD lymphocytes [108]; however, in mitochondria isolated from AD platelets, the activity of complex IV, or cytochrome c oxidase, is significantly decreased [109,110,111], indicating differences in the stage of disease at which the samples were obtained or differences in cellular responses. The activity of aconitase is significantly decreased in mitochondria from both MCI and AD lymphocytes [112].

In addition to the redox proteomics results described above that identified proteins with elevated markers of oxidative and nitrosative modifications to proteins in AD brain or models thereof such as protein carbonyls, 3-NT, and protein-bound HNE, another important index of protein nitrosative modification is S-nitrosylation. Nitric oxide-mediated reactions with intermediate oxidation states of Cys thiol moiety lead to nitrosylation of the S-atom of this amino acid in proteins. Most often, such modifications inhibit protein functions. Selected studies of the S-nitrosylation proteome in AD identified key proteins with altered functions associated with antioxidant, glycolysis, mitochondrial export, calcium homeostasis, synapses, apoptosis, mitochondrial dynamics, protein folding, and neuroinflammation, among other functions [113,114,115,116]. Many of these proteins are the same as those modified by other indices of oxidative and nitrosative stress noted above. This observation is consistent with the notion that specific proteins are more vulnerable to oxidative and nitrosative damage in the AD brain than other proteins.

There has been a long-standing debate as to whether oxidative stress, including damage indexed by protein oxidation and lipid peroxidation, is involved in the mechanisms leading to AD or consequences of AD neuropathology and altered neurochemistry and neurophysiology [117]. In our opinion, this is not a case of “either or”, but rather, “both and”. Aβ42 oligomers cause oxidative damage as mentioned above, and these oligomers also lead to synaptic dysfunction correlated with cognitive loss [8,9,26]. Mitochondria, too, are damaged by oxidative and nitrosative stress in AD as noted [34].

More research is needed to understand the impact of increased protein oxidation and lipid peroxidation on the mitochondrial proteome, and a focus on mitochondria from peripheral tissues may lead to the identification of potential biomarkers that are so desperately needed.

## 6. Implications of Mitochondrial Oxidative and Nitrosative Stress in MCI and AD: Correlations with Imaging Studies and Targeted Therapies

The human brain comprises approximately 2% of body mass but consumes approximately 20% of daily oxygen intake [118]. In the brain, ATP is required for neurotransmitter synthesis, protein turnover, neurotransmission, transport, and maintenance of ion gradients [119]. The high metabolic need of the brain cannot be met by glycolytic activity. Rather, neurons rely on the tricarboxylic acid cycle and oxidative phosphorylation housed in mitochondria to produce ATP [72,120]. However, oxidative stress, mitochondrial dysfunction, and glucose hypometabolism have been well documented in MCI and AD brain [8,53,74,75,76]. Oxidative modification and the decreased activity of several key glycolytic enzymes in MCI and AD brain are consistent with the observed glucose hypometabolism [35]. While glucose is a preferred fuel of the central nervous system, lactate and ketone bodies such as 3-β-hydroxybutyrate and acetoacetate can be utilized by neurons [121]. Unlike glucose metabolism, ketone metabolism does not appear to be altered in the early progression of AD, as ketones are used at the same rate in MCI and early AD as in the control brain [122,123]. Further research is needed to determine if ketone supplementation is beneficial.

Astrocytes express glucose-6-phosphatase-β (G6Pase-β) and are thus capable of gluconeogenesis, or the synthesis of glucose from non-carbohydrate precursors such as aspartate, glutamate, alanine, and lactate [124]. This suggests that gluconeogenic activity in astrocytes could function to build up a glucose concentration gradient to boost the flow of glucose from astrocytes to neurons [125]. Under oxidative stress, glucose in the brain is not only needed to produce ATP, but it would be diverted to produce much needed NADPH via the pentose phosphate pathway to replenish thiol-containing antioxidants such as thioredoxin, peroxiredoxin, and glutathione, as the oxidative reactions that produce NADPH are irreversible and require glucose-6-phosphate as a substrate [126]. A recent study demonstrated apoE4 isoform-specific differences in astrocytic glucose utilization in vitro. Compared to the apoE2 or apoE3 isoforms, astrocytes expressing the apoE4 isoform exhibited increased lactate synthesis, pentose phosphate pathway flux, and gluconeogenesis [127]. Further study is necessary to determine if these isoform-specific differences in glucose utilization occur in vivo.

Neurons could also use glucogenic or even ketogenic amino acids to generate TCA intermediates to produce ATP through oxidative phosphorylation when glucose is scarce [128]. Glutamate and glutamine are the most abundant amino acids in the human brain [129]. Glutamate is produced from the transamination of α-ketoglutarate where branched-chain amino acids likely serve as the amine donor resulting in the formation of their respective α-keto acids, which could be further catabolized to yield propionyl-CoA, succinyl-CoA, and acetyl-CoA [130]. Glutamine is produced from glutamate by the activity of glutamine synthetase (GS) [124,131]. Glutamate functions as an excitatory neurotransmitter and a glutamate–glutamine cycle exists between astrocytes and neurons, allowing for the movement of anaplerotic carbon units and the sequestration of ammonia, which is neurotoxic [132]. However, the use of such amino acids as a compensatory energy supply is likely not a long-term solution. Amino acid levels can become quickly depleted, and the oxidative modification of necessary enzymes and transporters could diminish the ability of neurons to utilize these alternative energy sources. The glial glutamate transporter (GLT-1), also known as the excitatory amino acid transporter-2 (EAAT2), is responsible for the uptake of synaptic glutamate, but its activity is decreased in AD brain, which is likely due to oxidative modification by HNE [26,131]. GS is also oxidatively modified in both MCI and AD brain [44,54,59], likely resulting in the significantly decreased activity observed in AD brain [133,134]. Metabolomic studies of MCI and AD CSF and plasma report altered levels of amino acids compared to controls, indicating that alterations in amino acid metabolism are present in AD [135,136]. More research is necessary to understand the complex network of metabolic perturbations in AD.

**Figure 4:**
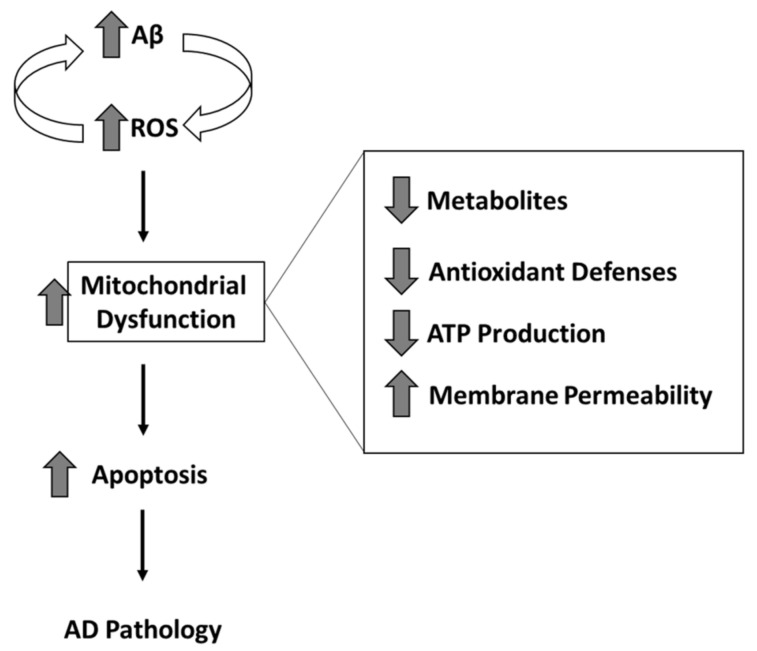
Mitochondrial dysfunction related to oxidative and/or nitrosative stress that is in turn associated with amyloid β-peptide oligomer inserted in the mitochondrial membranes. Consequences of these oxidative or nitrosative stress-mediated dysfunction include, among others, decreased levels of ATP production, decreased levels of metabolites, decreased levels of antioxidant defense, and an increased mitochondrial permeability transition pore (MPTP) transport of cytochrome c, all of which are highly detrimental to both mitochondria and consequently neurons. See text for more details.

Efforts continue to be placed on the early detection of AD as changes in brain chemistry begin to occur 20 years or more prior to the onset of noticeable cognitive symptoms [137,138,139,140]. A recent longitudinal study of asymptomatic autosomal dominant carriers of AD provided further insight into the progression of brain chemistry changes with Aβ deposition occurring first, followed by hypometabolism, and finally structural atrophy [141]. These observations are consistent with our hypothesis that Aβ-oligomer-induced oxidative stress impairs glucose metabolism, contributing to mitochondrial dysfunction, leading to synaptic dysfunction, and eventually resulting in neuronal death (Figure 4) [8,142]. While the deposition of Aβ occurs early in the progression of the disease [141], oligomeric Aβ rather than fibril Aβ is regarded as the toxic species of this peptide [143,144,145,146]. Oligomeric Aβis a precursor to fibrillar Aβ and this hydrophobic peptide, unlike fibrils, is small enough to solubilize in lipid bilayers. Accordingly, membrane-resident oligomeric Aβ is present and induces oxidative damage (lipid peroxidation) prior to the detection of Aβ deposition [8], which is consistent with the observation that the disease is initiated decades before the onset of cognitive symptoms. The presence of Aβ oligomers in mitochondrial membranes is consistent with Aβ-induced mitochondrial dysfunction occurring early in the disease progression timeline [34,53]. Consequently, there is growing evidence that interventions to prevent or delay the onset of AD need to occur decades prior to the onset of cognitive symptoms.

Based on the hypothesis that Aβ-induced oxidative damage to mitochondria is an early event in the progression of AD, mitochondria have been targeted for the study and development of therapeutics [53,147,148]. This is still a rather nascent field of investigation, and further studies are needed. Toward this goal, a recent compilation of mitochondria-targeted therapeutic strategies for AD was published [148]. Among these mitochondria-targeted therapeutic agents are those that target mitochondrial: bioenergetics; glucose metabolism; biogenesis; uncoupling proteins; mitophagy; and oxidative stress. In keeping with the theme of this review, we discuss the latter. Alpha-lipoic acid is a cofactor for pyruvate dehydrogenase and alpha-ketoglutarate dehydrogenase, which are key components in glucose metabolism; when given to dogs and rodents, it decreases oxidative damage in the brain and improves cognition [149,150]. In a small clinical pilot study, probable AD patients given alpha-lipoic acid and two choline acetyltransferase (CAT) inhibitors had slower cognitive decline than probable AD patients given only the two CAT inhibitors [151]. Another mitochondria-targeted therapeutic agent is coenzyme Q10 (CoQ10), which is a key participant in the Q-cycle of electron transport in the inner mitochondrial membrane. CoQ10 led to decreased Aβ pathology in aged transgenic mice with human AD *presenilin-1* mutation, and in a different study, a quinone-containing derivative of CoQ10 (MitoQ) that targets mitochondria, improved memory, and neuropathology in a transgenic mouse model of AD [152,153]. Continued development of mitochondria-targeted therapeutic agents will be important for strategies to slow or stop the progression of AD. 

While age, chromosomal sex, family history, and *apoE* genotype remain the primary risk factors for the development of AD [154,155], there are several controllable risk factors including obesity [156,157], hyperlipidemia [158,159], hypertension [160,161,162,163], type 2 diabetes [164,165,166,167], and sedentary lifestyle [168] that can be addressed in an effort to decrease the risk of developing AD. Mitochondrial oxidative stress is increased by the consumption of excess calories and, in turn, it leads to mitochondrial DNA damage [120], which has been described in an AD brain [169,170] in which base deletions correlate with complex IV deficiency in an AD hippocampus [171]. Collectively, this suggests that eating a heart-healthy diet [172,173,174,175,176] and increasing physical activity [177,178,179,180] could delay the onset of AD. The Finnish Geriatric Intervention Study to Prevent Cognitive Impairment and Disability (FINGER) multidomain lifestyle intervention trial found that interventions in nutrition, exercise, cognitive training, and management of metabolic and vascular risk factors benefitted an at-risk population [181], even in *APOEε4* carriers [182]; however, no significant differences in brain volume or cortical thickness were determined by MRI after two years of intervention [183]. This suggests that two years is not enough or that the intervention, while improving cognitive processing speed, is too late to impact brain volume and may be more effective if implemented earlier in life. Given the similarity in mitochondrial dysfunction observed in obesity, type 2 diabetes, and AD [184,185,186,187], it is reasonable to hypothesize that these lifestyle interventions, if implemented in midlife or earlier, may protect against the mitochondrial dysfunction evident in AD.

## 7. Concluding Remarks

Alzheimer disease represents a worldwide public health crisis that is expanding due to improvements in health care that permits increased longevity among people. The economic and personal costs are staggering already, and the implications for the future are highly daunting. Consequently, new insights into ways to slow the progression of AD are a high priority among nations of the world.

Important components of the pathogenesis and progression of AD are oxidative and nitrosative stress in brain. Mitochondria provide reactive oxygen species that contribute to both oxidative and nitrosative stress and are themselves targets of these free radical-mediated processes. Consequently, we posit that better understanding of the molecular processes that lead to mitochondrial oxidative and nitrosative stress potentially will lead to selective therapeutic targets in mitochondria that slow or retard the progression of AD.

In this review, we trust that we have increased interest in the AD research community for investigations designed to gain additional much-needed understanding of the molecular basis of AD that, we assert, ought to include studies of mitochondria in this devastating disorder.

## Figures and Tables

**Table 1:** Summary of oxidatively modified mitochondrial proteins identified by redox proteomics and enzymatic activity in mild cognitive impairment (MCI) and Alzheimer disease (AD).

Protein	Oxidative Modification	Disease State	Brain Region	Activity	Reference
Aconitase	H	Late AD	Hippocampus	↓	[54]
MDH	H	Early AD	IPL	↑	[55]
LDH	H	MCI	Hippocampus	↓	[56]
ATP Synthase	H	MCI	Hippocampus	↓	[56]
	H	MCI	IPL	↓	[56]
	H	Early AD	IPL		[55]
	H	Late AD	IPL	↓	[54]
	N	Late AD	Hippocampus		[57]
MnSOD	H	Early AD	IPL	↓	[55]
	H	Late AD	IPL		[54]
EF-Tu	H	MCI	IPL		[56]
HSP70	H	MCI	Hippocampus		[56]
	N	MCI	Hippocampus		[58]
	C	MCI	IPL		[59]
HSP90	C	MCI	IPL		[60]
VDAC1	N	Late AD	Hippocampus		[57]

C, carbonylation; N, nitration; H, HNE bound.
